# Cohort profile: The Golden Retriever Lifetime Study (GRLS)

**DOI:** 10.1371/journal.pone.0269425

**Published:** 2022-06-09

**Authors:** Julia Labadie, Brenna Swafford, Mara DePena, Kathy Tietje, Rodney Page, Janet Patterson-Kane

**Affiliations:** 1 Scientific Programs Department, Morris Animal Foundation, Denver, Colorado, United States of America; 2 Flint Animal Cancer Center, Colorado State University, Fort Collins, Colorado, United States of America; University of Bologna, ITALY

## Abstract

The aim of this article is to provide a detailed description of the Golden Retriever Lifetime Study (GRLS), a prospective cohort study investigating nutritional, environmental, lifestyle, and genetic risk factors for cancer and other common diseases in dogs. Primary outcomes of interest include hemangiosarcoma, lymphoma, osteosarcoma, and high-grade mast cell tumors. Secondary outcomes of interest include other cancers, hypothyroidism, epilepsy, atopy, otitis externa, hip dysplasia, heart failure, and renal failure. A total of 3,044 United States Golden Retrievers aged 6 months to 2 years completed baseline enrollment from June 2012 to April 2015. As of May 31, 2021, 2,251 dogs remain engaged in the study, 352 have died, and 441 are lost to follow-up. Extensive annual questionnaires completed by owners and veterinarians gather information about lifestyle, environmental exposures, physical activity, reproductive history, behavior, diet, medications, and diagnoses. Dogs also have annual veterinary examinations and biospecimen collection (blood, serum, hair, nails, feces, urine) for biobanking. Additional reporting, including histology and tumor biobanking, is conducted for any malignancies or deaths. When an animal dies, full medical records are obtained, and necropsies are requested at owner discretion. Full or partial necropsies have been performed on 218 dogs. Questionnaire data are freely available to researchers with approved credentials who agree to a data use agreement. In addition, researchers can submit proposals to utilize biospecimens or obtain additional data.

## Why was the cohort set up?

According to the American Veterinary Medicine Association, the 2016 United States dog population was 76.8 million, with 48.3 million households owning a dog [1]. Cancer is a significant cause of canine morbidity and mortality and is thought to result in the death of approximately one in four dogs, with multiple breeds having an increased risk of developing one or more specific cancer types [2, 3]. Dogs are increasingly being used as natural models for human diseases, especially certain cancers, due to morphological and clinical similarities [4]. Benefits of canine models include: 1) reduced genetic variation within dog breeds [5–7], enhancing the ability to detect genetic risk factors for disease, 2) similar environmental exposures as their human counterparts, 3) shorter lifespan, enabling researchers to follow dogs from birth to death in <20 years.

Leveraging these benefits, the Golden Retriever Lifetime Study (GRLS), a longitudinal cohort study, was developed. A specific dog breed was chosen to limit genetic variability, facilitate the study of non-genetic factors, and enhance the potential of obtaining breed-relevant wellness strategies. Golden Retrievers are repeatedly ranked in the top five most popular breeds by the American Kennel Club; this holds across states, meaning that the environments where they reside are expected to be relatively diverse. They generally score highly for being ‘happy’ and trainable i.e., likely amenable to annual, complex veterinary visits [8, 9]. In addition, this breed is highly prone to cancer; a survey of 1,444 Golden Retrievers conducted by the Golden Retriever Club of America in 1998 indicated that cancer caused 61% of the 420 deaths with the most common types being hemangiosarcoma, lymphoma, mast cell tumor, and osteosarcoma [9]. Cancer-related mortality of 65% was documented in a recent necropsy study of 652 Golden Retriever dogs at a USA veterinary academic hospital, with the most common diagnosis being hemangiosarcoma, followed by lymphoid malignancies [10]. Lower cancer rates among golden retrievers (20–39%) were documented in studies in the UK and Scandinavia among populations registered with kennel clubs or enrolled with pet insurance companies [11–13]. Direct comparisons across different cohorts have not yet been performed but will likely help address concerns that limit the generalizability of single cohort studies, including selection bias.

GRLS is managed and funded by the Morris Animal Foundation (MAF), a non-profit organization based in Denver, Colorado that invests internationally in science to improve animal health. The Foundation leadership recognized the importance of a large-scale cohort study to track individual dogs for identification of genetic, environmental and lifestyle risk factors that contribute to cancer and other significant diseases, and their capacity to set up, fund and maintain a study of this type. Cohort studies of companion animals have historically been very limited due to the cost and time involved and complexities of access to and communication with participating owners.

The aim of GRLS is to evaluate nutritional, environmental, lifestyle, reproductive, and genetic risk factors for cancer and other common disorders in the golden retriever breed [8]. The primary study objective is to document and collect data on 500 dogs diagnosed with the primary endpoint cancers: hemangiosarcoma, lymphoma, osteosarcoma, and high-grade mast cell tumors. However, information is collected on all conditions diagnosed, to facilitate evaluation of other cancers and diseases commonly documented in golden retrievers such as hypothyroidism, osteoarthritis, and allergies. Datasets and biological sample repositories are available for current and future analyses.

The objective of this Cohort Profile manuscript is to provide a comprehensive description of the GRLS cohort, including an overview of the study population, data collection, participant characteristics, peer-reviewed publications to date, and availability of datasets and biological samples to the scientific community.

## Cohort description

In this observational, population-based cohort, owners of 3,044 golden retrievers aged six months to two years residing in the contiguous United States were recruited for participation from 2012 to 2015. Analysis of cohort data and samples is managed either by the Foundation or through extramural proposals or contracts. Details of study recruitment, inclusion and exclusion criteria have been previously published [8, 14]. Briefly, owners were recruited via advertisements with the Golden Retriever Club of America, Golden Retriever Foundation, and regional golden retriever clubs, veterinary professional organizations, as well as social media networks.

Owners and dogs were screened for enrollment by completing an owner profile with demographic and registration information. Enrollment was stratified by five geographical regions (Pacific, Mountain, Midwest, Northeast, and South) as well as dog sex and spay/neuter status. Dogs were required to have a three-generation pedigree. Once dogs were screened for enrollment, invited owners completed written informed consent. They subsequently completed the annual owner questionnaire, veterinarian visit, and sample collection. Veterinarians confirmed the dogs were free of life-limiting conditions and completed written informed consent for their participation in the study prior to completing the annual veterinarian questionnaire. The study protocol was approved by Morris Animal Foundation’s Animal Welfare Advisory Board.

## What has been collected?

### Annual data collection

**Fig 1:**
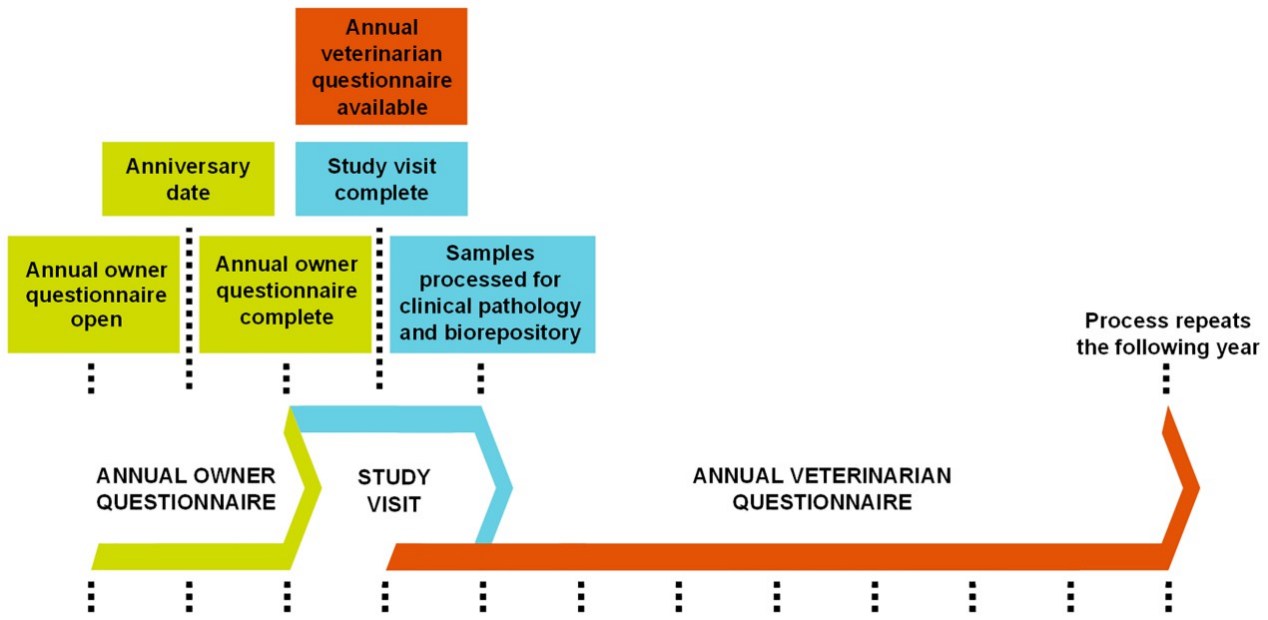
Study timeline and components. Three study components are conducted annually around the time of the dog’s anniversary date: 1) Annual Owner Questionnaire, 2) annual veterinary visit (‘‘Study Visit”), and 3) Annual Veterinarian Questionnaire. The Annual Owner Questionnaire is available from one month prior to the anniversary date until 10 months after the anniversary date, but most owners complete it within one month of the anniversary date. Once the owner completes the questionnaire, they are sent a sample collection kit and asked to schedule their Study Visit. At the Study Visit, a full physical examination is performed and core samples are collected for clinical pathologic processing and biorepository storage. The typical time between Annual Owner Questionnaire completion and Study Visit is one month. The veterinarian can complete the Annual Veterinarian Questionnaire any time after the Study Visit is completed. There is no requirement surrounding this time frame, but veterinarians are encouraged to fill it out as soon as they are able, ideally prior to the next Study Visit.

Three study components are conducted annually: owner questionnaire, veterinarian examination and questionnaire, and sample collection. The Annual Owner Questionnaire is available from one month prior to the anniversary date until 10 months after the anniversary date (Fig 1), but most owners complete the questionnaire within one month of their anniversary date. The comprehensive Annual Owner Questionnaire solicits information on each dog’s lifestyle, travel history, reproductive history, physical activity, over-the-counter medications, flea, tick, and heartworm preventives, at home dental care, grooming history, diet and feeding practices, environment, living conditions and exposures, and a behavioural questionnaire (C-BARQ) [15]. Once the owner completes the questionnaire, they are sent a sample collection kit and asked to schedule their annual veterinarian visit (“Study Visit”). At the Study Visit, a full physical examination is performed and core samples (whole blood, serum, urine, feces, hair clipping, and toenails) are collected for clinical pathologic processing and biorepository storage. Veterinarians are provided with a comprehensive sample collection instruction manual The Annual Veterinary Sample Kit: Collection & Shipping Instructions is available at: https://www.morrisanimalfoundation.org/sites/default/files/filesync/GRLS-Annual-Kit-Instructions.pdf that details how to collect and submit each of our core samples. The veterinarian questionnaire The Annual Veterinarian Questionnaire is available at: https://www.morrisanimalfoundation.org/sites/default/files/filesync/GRLS-Annual-Veterinarian-Questionnaire.pdf includes data on each dog’s medical history, physical examination findings including height at withers, weight, and body condition score, a map of superficial masses, vaccination history, and prescription medication history. All questions are phrased to ask about diagnoses and medications in the 12 months preceding the Study Visit. Owners and veterinarians also report the sire and dam’s medical history, if known. The typical time between Annual Owner Questionnaire completion and Study Visit is one month. The veterinarian can complete the Annual Veterinarian Questionnaire any time after the Study Visit is completed. There is no requirement surrounding this time frame, but veterinarians are encouraged to fill it out as soon as they are able, ideally prior to the next Study Visit.

### Annual laboratory analysis and biospecimen banking

Core samples are shipped overnight to a biospecimen repository to be frozen and stored for subsequent analyses (samples are not flash frozen). Details on the amount collected and stored have been previously described [8] and are outlined in Table 1. At baseline, DNA was extracted from whole blood samples and stored for future analyses. We are currently in the process of genotyping all dogs for genome-wide association studies; this data will ultimately be made available for use by research scientists.

**Table 1:** Biorepository specimen summary as of May 31, 2021.

Specimen	Storage temperature	Amount collected at annual veterinary visit	Banked aliquots available to researchers	Total number of samples collected
Genomic DNA^^^	-80°C	N/A	DNA aliquots adjusted to 100 ng/ul	3,044
Whole Blood^^^	-80°C	10 mL	250 ul aliquots	18,974
Serum	-80°C	10 mL	250 ul aliquots	18,954
Urine	-80°C	5 mL	1 mL aliquots	18,965
Feces	-80°C	~1 gram	Not available	18,915
Hair Clippings	-20°C	2” long lock, ¼" in diameter	1 sample per visit	18,875
Nail Clippings	-20°C	5–10 clippings	1 sample per visit	18,885

^^^Genomic DNA was extracted and banked for the baseline study visit only; whole blood was banked at all subsequent visits

Additional samples of blood, serum, feces, and urine are overnighted to a veterinary diagnostic reference laboratory that performs a complete blood count, serum biochemistry profile, fecal evaluation for ova and parasites, urinalysis, heartworm antigen test, and thyroid hormone (total thyroxine (T4)) level. Results are shared with the study veterinarians and added to our database. Detailed information about laboratory tests and methodology are provided in S1 Table.

### Additional follow-up for malignancies and death

In addition to the annual follow-up, owners and veterinarians are instructed to contact us in the event of a suspected or confirmed malignancy diagnosis or death. In the event of a suspected malignancy, we send veterinarians a biopsy kit and request samples be submitted to our diagnostic laboratory for histology with, for lymphoproliferative disorders, the additional option of flow cytometry and polymerase chain reaction for antigen receptor rearrangement (PARR) conducted at the Colorado State University (CSU) Clinical Immunology Laboratory. Veterinarians are provided with a comprehensive sample collection instruction manual that details how to collect and submit samples for histopathology, flow cytometry, PARR, and biobanking. The addition of lymphoma subtyping in September 2019 has led to a high percentage (79%) of dogs with immunophenotype data available. When possible, we also request a 5 mm^3^ tumor sample be submitted to our biorepository in RNALater. Once a malignancy is confirmed, we request veterinarian completion of an additional questionnaire to obtain details about diagnostics performed and actions taken.

A necropsy is encouraged, but not required, when a dog dies. A death and necropsy questionnaire is requested from the registered Study veterinarian, the last veterinarian who saw the dog, and the veterinarian conducting the necropsy (if applicable) to obtain information about cause and manner (i.e., euthanasia versus natural death) of death as well as gross necropsy findings if available. When possible, necropsies are performed using standardized kits and a sample collection manual provided to the veterinarian. We request the veterinarian collect samples of both diseased and healthy liver, kidney, adrenal, spleen, lymph node, heart, thyroid and parathyroid as well as samples of any suspected malignancies. Tissues are collected in formalin for histopathology and in RNALater to be submitted for biobanking. If the necropsy is performed at a diagnostic laboratory or by a willing veterinarian, we also request haired skin sample, eyes, lung, oesophagus, stomach, duodenum with pancreas, jejunum, ileocecocolic junction, urinary bladder, skeletal muscle, bone, bone marrow, synovial fluid, and brain and/or spinal cord. Additionally, we request collection of core samples and samples of any effusion for laboratory analysis and biobanking at the veterinarian’s discretion. Our operations team collects full medical records, including the primary veterinarian and any specialist records, after a dog dies. These records are used to adjudicate cause of death and any major diagnoses as needed.

We strive for all histologic biopsy and post-mortem specimens to undergo an adjudication process involving two to three independent pathologists. After the initial diagnostic laboratory completes their review, specimens are transferred to the CSU Veterinary Diagnostic Laboratory. A CSU pathologist completes an independent review of the specimens, blinded to any previous diagnoses. The findings are reported to MAF and reviewed by the operations team. If the two pathologists agree, the case is considered adjudicated and assigned a tier of confidence of one. Should the secondary read disagree with the initial laboratory, a third pathologist completes a blinded independent review of the specimens. If two out of the three diagnoses agree, the case is considered adjudicated, and the conflicting diagnosis is overruled. Should the initial, secondary, and tertiary read all disagree, the pathologists convene to come to a consensus diagnosis. Additional immunohistochemical stains are employed as needed. Paraffin wax blocks and histology slides from adjudicated cases are transferred to MAF, where they are inventoried and stored.

Not all malignancies are diagnosed histologically due to cost constraints, invasiveness of sampling, or owner preferences. Tiers of confidence have therefore been assigned for all malignancy diagnoses (S2 Table). Tier 1 represents a definitive diagnosis microscopically confirmed via histology or cytology, read by a board-certified pathologist. For lymphoproliferative disorders, flow cytometry or PARR are also accepted. Tier 2 represents a presumptive diagnosis based on direct visualization or imaging without microscopic confirmation (e.g., a dog with a pericardial effusion and a mass visualized on the right atrium would be assigned tier 2 hemangiosarcoma). For a diagnosis to be considered Tier 2, imaging must be performed by an appropriate board-certified veterinary specialist (i.e., radiographs read by a board-certified veterinary radiologist). In-house cytology (i.e., cytology read by the attending veterinarian) is also considered a Tier 2 diagnosis. Tier 3 represents a presumptive diagnosis based on clinical suspicion only (e.g., a dog with lymphadenopathy where the owner declined diagnostics and the veterinarian listed lymphoma as a differential diagnosis would be a Tier 3 lymphoma).

### Study changes over time

Two major operational changes have occurred since the study’s inception: a change in the study’s data management system and a change in the primary diagnostic laboratory. In July 2020, we transitioned from a third-party study management company to in-house operations. This involved 1) starting an in-house call center for all participant and veterinarian communications, 2) building and shipping all specimen collection kits, and 3) creating a custom database for study oversight, questionnaire completion, and data analytics.

In December 2020, we changed diagnostic laboratories. Details comparing the instruments used and analytes tested for each laboratory are available in S1 Table. Prior to transitioning laboratories, we conducted a 1 month, 100 dog bridging study to evaluate whether laboratory results were comparable across the two diagnostic laboratories. Overall, results were comparable, supporting our decision to move forward with the change.

Changes to the questionnaires over time have predominately been minor, including clarifying questions participants found confusing, soliciting additional details, changing from Hill’s Body Fat Index (Hill’s Pet Nutrition, Topeka, KS) to the 9-point Purina Body Condition Score (Purina Animal Nutrition, St. Louis, MO) after the baseline questionnaire, and expanding checkbox diagnosis options for veterinarians. The transition to in-house operations has enabled more rapid, flexible questionnaire changes, resulting in a few major study additions. First, we changed from checkbox diagnoses to using SNOMED Clinical Terms (https://www.snomed.org/) to allow for a broader range of diagnoses while still having controlled terminology. Second, we recently launched the “Golden Age Study” in partnership with Elanco Animal Health and the Purina Institute. This optional twice-yearly study addition includes previously validated questionnaires to assess osteoarthritis (the Liverpool Osteoarthritis in Dogs (LOAD)) [16] questionnaire for owners and the Canine OsteoArthritis Staging Tool (COAST) [17] for veterinarians and the DISHAA (Disorientation, social Interactions, Sleep/wake cycles, House soiling, learning and memory, Activity and Anxiety) [18] tool to assess Cognitive Dysfunction Syndrome by both veterinarians and owners. Third, we added a death and necropsy questionnaire which allows veterinarians to enter health history changes between when the last annual veterinary questionnaire was completed and the date of death. In addition, gross necropsy findings (if performed) and suspected cause of death are collected. Historically, this information was largely captured by MAF staff veterinarians and veterinary assistants abstracting medical records, so these changes decrease MAF staff burden and improve data integrity.

## Study participation

Overall GRLS has had excellent participation and retention (S1 Fig). As of May 31, 2021, overall retention is 86% (including 2,251 dogs enrolled and alive, 352 enrolled deceased), with only 441 dogs lost to follow-up. Reasons for active withdrawal were gathered for 96 (of 441) dogs; the most common reasons included owner hardship (n = 28; 29%), rehoming the dog (n = 20; 21%), and the dog having high anxiety at the veterinarian (n = 14; 15%).

The annual participation rate, defined as completion of all three study components (annual owner questionnaire, Study Visit with biospecimen collection, and annual veterinarian questionnaire) has ranged from 74% to 87% for study years 1 through 5. Since completion of the annual veterinarian questionnaire is contingent on completion of the annual owner questionnaire, we see slightly lower overall participation by veterinarians (annual owner questionnaire ranged from 80% to 93% for study years 1 through 5, annual veterinarian questionnaire completion ranged from 75% to 89%). However, if we consider only veterinarians with the opportunity to complete an annual veterinarian questionnaire as the denominator (I.e., number of participants with a complete annual owner questionnaire), the annual veterinarian questionnaire completion rate ranged from 92 to 96% for study years 1 through 5. Currently, we have banked nearly 19,000 of each sample type we collect (Table 1).

Demographic information for the cohort as of their last questionnaire date are shown in Table 2. Dogs remaining in our cohort are, on average, 8.3 years old (range, 6.7–11.3 years) as of May 2021. Approximately 19% (n = 418) are still intact, with a relatively even distribution of age at gonadectomy (14% ≤ 6 months, 27% 6 months– 1 year, 20% 1–2 years, 21% >2 years). Geographic distribution remains consistent with our baseline recruitment, approximately evenly split between five regions. Most dogs are a healthy body condition score (Purina BCS of 4–5), but 37% are classified as overweight (BCS 6–7) or obese (BCS 8–9). Most dogs who were lost to follow-up were lost early in the study; as such, they were younger with a higher percent intact.

**Table 2:** Demographic information of study participants as of May 31, 2021.

	Current Enrolled (n = 2,251)		Dead (n = 352)		Lost to follow-up (n = 441)	
	n	%	n	%	n	%
Age at enrollment, years^^^	1.20	(0.47–2.94)	1.46	(0.41–2.98)	1.33	(0.52–3.25)
Current age, years^^^	8.26	(6.69–11.31)	7.28	(0.68–10.70)	2.78	(0.51–7.53)
Age at spay/neuter, categorical						
<= 6 months	313	14%	45	13%	53	12%
6 months—1 year	615	27%	98	28%	105	24%
1–2 years	450	20%	67	19%	43	10%
2–5 years	259	12%	26	7%	17	4%
> 5 years	196	9%	13	4%	1	0%
Intact	418	19%	103	29%	222	50%
Sex						
Female intact	131	6%	39	11%	106	24%
Female spayed	994	44%	120	34%	114	26%
Male intact	287	13%	64	18%	116	26%
Male neutered	839	37%	129	37%	105	24%
Geographic location						
Pacific	306	14%	43	12%	76	17%
Mountain	308	14%	51	14%	47	11%
Midwest	545	24%	78	22%	94	21%
Northeast	472	21%	69	20%	82	19%
South	620	28%	111	32%	142	32%
Body condition score^#^						
Underweight	16	1%	2	1%	6	1%
Normal	1198	53%	197	56%	283	64%
Overweight	738	33%	122	35%	138	31%
Obese	82	4%	12	3%	14	3%
Missing	217	10%	19	5%	0	0%

*All data are based on last owner- and veterinarian-completed questionnaire; age calculated as of 5/31/2021 for enrolled dogs, otherwise based on date of death/withdrawal

^median (range); age at enrollment is based on time of completion of all study components and thus may be higher than 2 years due to delays in completing the veterinary questionnaire

^#^Body condition score was missing for some dogs due to an error in the questionnaire when transitioning to the in-house database; this has been fixed for ongoing data collection

## Study endpoints

**Fig 2:**
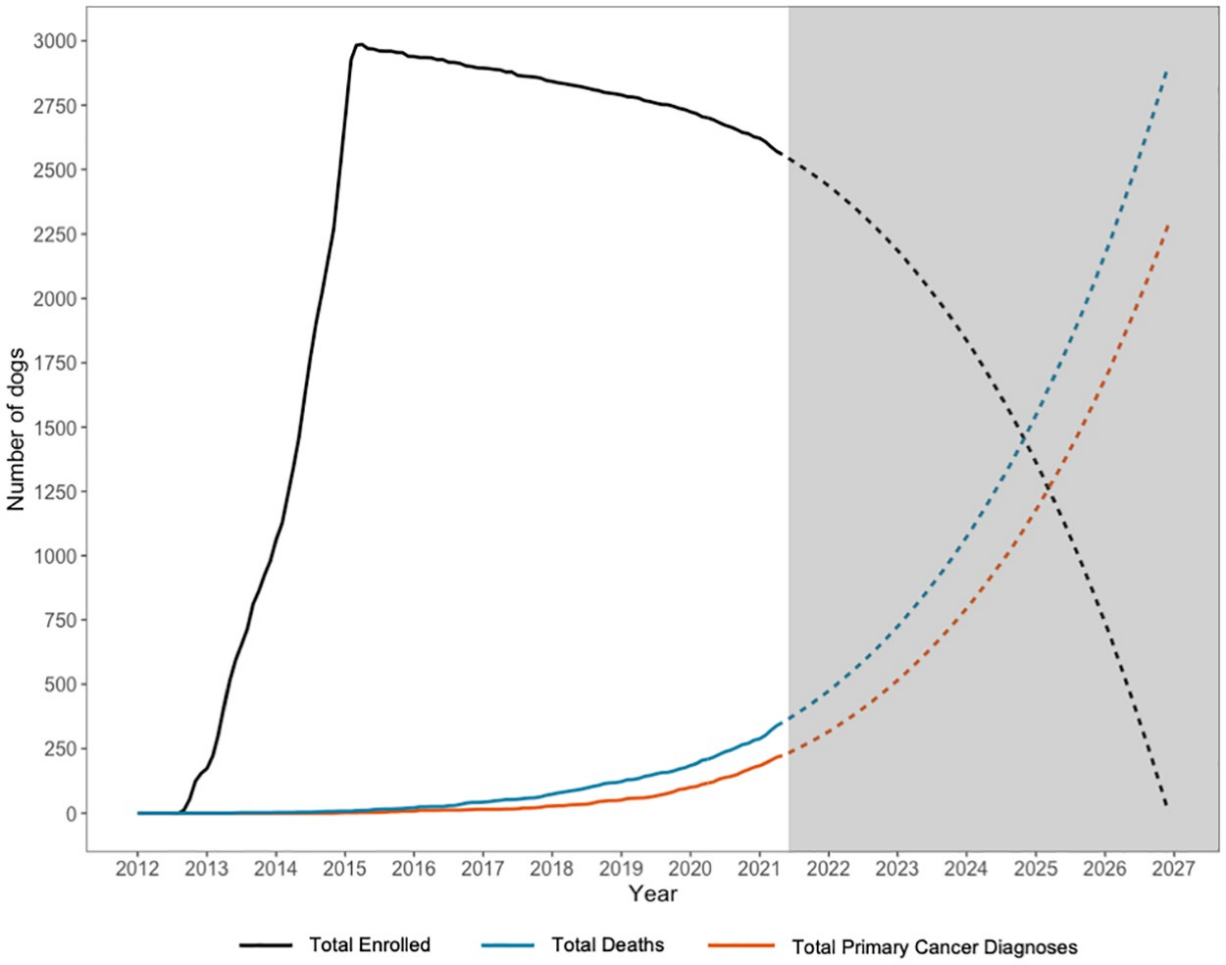
Observed and estimated enrollment, deaths, and primary cancer diagnoses. Projected data was created by fitting a cubic model of months since enrollment to observed data and projecting values to future months. Grey shading indicates the time period for projected data.

As of May 31, 2021, we have obtained 223 of 500 primary endpoints (45%). Hemangiosarcoma is the most common primary endpoint (n = 120), followed by lymphoma/leukemia (n = 85). There have been fewer diagnoses of high-grade mast cell tumors and osteosarcoma than expected, with only 10 and 8 cases, respectively. Most of these diagnoses fall into our tier 1 category of definitive diagnosis via histology or cytology (89 hemangiosarcoma [74%], 77 lymphoma/leukemia [90%], 9 mast cell tumors [90%], 6 osteosarcoma [75%]) (S2 Fig). Based on our current data, we estimate we will reach 500 primary endpoints around January 2023 (Fig 2).

Seventy-nine percent of lymphoma/leukemia cases were subtyped (n = 68) by various combination of IHC, flow cytometry, and PARR. These were evenly split between B- and T-cell subtypes (n = 30 and 32, respectively). B-cell subtypes included Diffuse Large B-cell Lymphoma (n = 22), undefined B-cell lymphoma (n = 7), and Marginal Zone lymphoma (n = 1). T-cell subtypes included Peripheral T-cell Lymphoma (n = 14), T zone lymphoma/leukemia (n = 7), and undefined T-cell lymphoma (n = 11). Additionally, there were 4 cases of acute leukemia, one epitheliotropic lymphoma, and one null-cell lymphoma.

**Fig 3:**
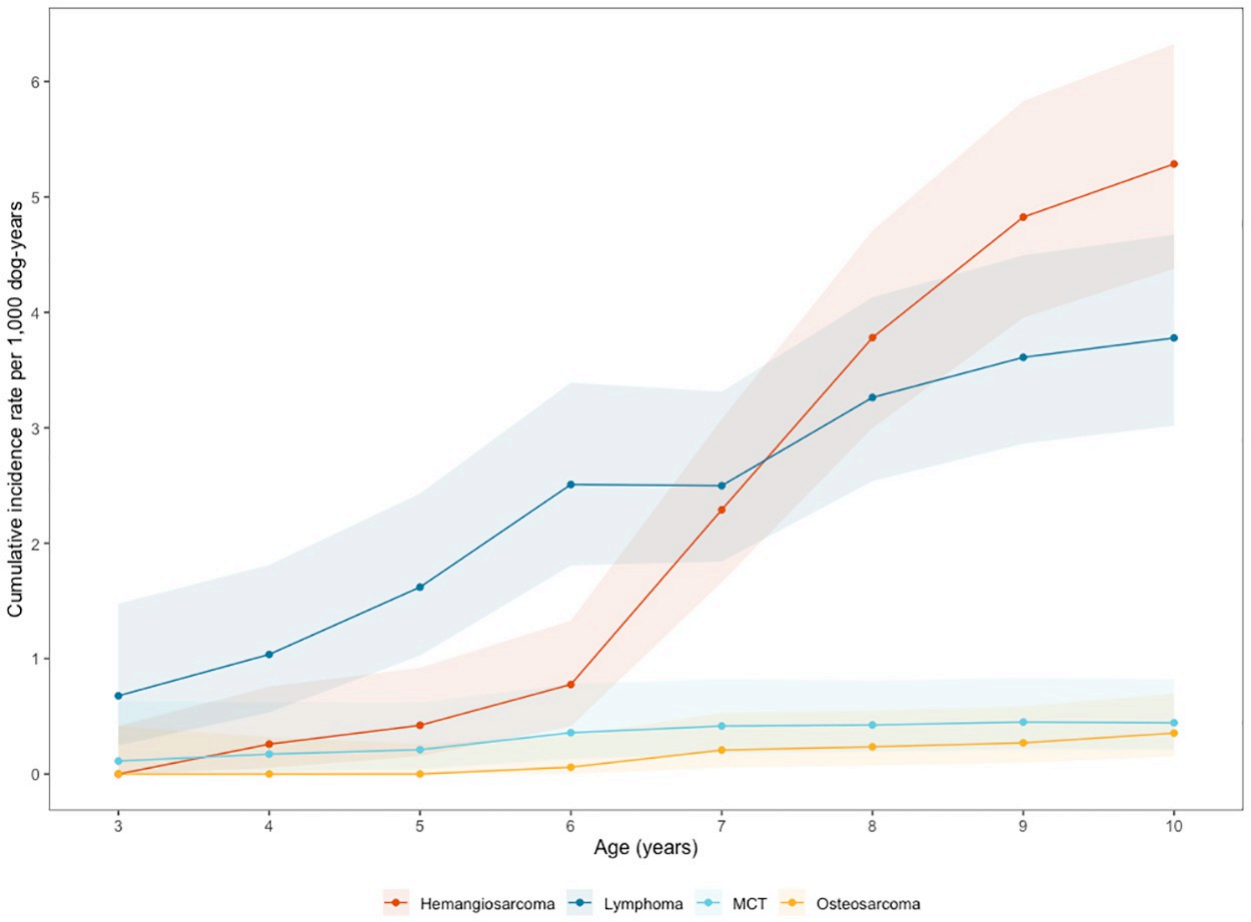
Cumulative incidence of the four primary endpoints. Cumulative incidence is shown by dog age in years and the shaded areas represent 95% confidence intervals. All diagnostic tiers (1–3) are included in incidence calculations.

The cumulative incidences by age have been documented for each of the primary endpoints (Fig 3). Lymphoma/leukemia was the most common cancer among dogs less than 6 years of age, showing a steady incidence over time. In contrast, hemangiosarcoma incidence was initially low, but grew steeply after age 6, becoming the most common cancer around age 8. As above, incidences of high-grade mast cell tumors and osteosarcoma have remained low.

To date, 352 dogs have died; 70% of these deaths (n = 248) were attributed to cancer. Nearly two-thirds of those dogs (n = 218; 62%) have had a necropsy. The proportion of dogs receiving a necropsy has increased over time due to increased participant outreach and clarification of the necropsy protocol (65% of deceased dogs have had a necropsy in the past two years vs. 55% prior). Based on our observed participant death rate trend, we estimate most participants will be deceased around January 2027 (Fig 2).

## Key findings and publications

To date, there have been six peer-reviewed articles published using GRLS data that can be found at: https://datacommons.morrisanimalfoundation.org/publications [8, 14, 19–22]. Details of select findings are outlined below.

### Biochemistry variation in healthy dogs

Biochemistry analyte annual change intervals were calculated in a subset of 190 healthy study participants and validated in an independent sample of 238 healthy study participants [21]. This study highlights biologic variability in wellness testing and the utility of obtaining baseline laboratory data to assess individual variation.

### Factors associated with owner compliance

A study conducted to evaluate factors associated with owner non-compliance after the baseline study visit [20] found that non-compliant owners were more likely to have dogs with no vaccination status and dogs who slept in the garage versus bedroom. This information may be useful for predicting compliance in future cohort studies or in targeted recruitment and oversampling of groups less likely to be compliant. Future analyses will evaluate whether these factors are consistent predictors of compliance at later time points.

### Age at gonadectomy

Gonadectomy at 6 months or younger was found to be associated with an increased risk of orthopaedic injury [22] In addition, gonadectomy at any age increased the risk of being overweight or obese. This manuscript, along with other corroborating publications [23–25], has contributed to the growing discussion on the need for appropriate breed-specific recommendations on optimal gonadectomy ages for dogs.

### Inbreeding depression

In a subset of 100 female intact dogs a statistically significant negative correlation was found between a genomic measurement of inbreeding and fecundity [19]. This indicates that golden retrievers would benefit from practices that limit inbreeding and paves the way for investigation of other affected traits.

### Overview publications

Two additional publications provide an overview of the study. The first publication highlighted the motives, goals, and design of the study prior to completing enrollment [8]. The second publication described baseline population characteristics among enrolled dogs [14].

## What are the main strengths and weaknesses?

The GRLS is the largest, lifetime comprehensive veterinary cohort study in the United States that provides unique data and sample access for basic and translational research. Comprehensive information is available for each dog, including diagnostic laboratory testing results, medications, veterinary diagnoses, and environmental and lifestyle data. With the longitudinal design and a combination of questionnaire data and biological samples, we are well-equipped to evaluate biomarkers for early disease detection.

Our overall participation and retention rates are noteworthy. The Annual Veterinary Questionnaire completion rate is slightly lower than the Annual Owner Questionnaire completion rate due to the order of operations for Study components. Fortunately, much of this data can be recovered through obtaining medical records after the dog’s death. Future studies may consider abstracting medical records at regular intervals to obtain health and medication data.

Participating in GRLS requires a substantial commitment from owners. While we do not collect owner demographic data, it is likely that this is a select population of owners that may not be generalizable to all dog owners. Additionally, since we have limited our population to one dog breed and geographic area, some findings may not be generalizable to other breeds or regions. The aetiology of many diseases is not breed-specific and thus findings from this study are likely to benefit a broad range of dog breeds. However, there may be geographic differences in cancer incidence and prevalence, as suggested by some studies [11–13]. We suspect that our cohort will have a high degree of relatedness, which will be evaluated once we have completed genotyping all participants.

A major challenge going forward with this study includes adjudicating veterinarian diagnoses. As no specialized training is provided to participating veterinarians, the confidence of a veterinarian’s diagnosis and thus reporting on our annual questionnaires depends on many factors, including clinical experience, background and training, the owner’s willingness to pursue diagnostic testing, and practice norms. Adjudication is more straight-forward for cancer diagnoses since they are often accompanied by histology or other additional testing but can be more difficult for some of our secondary endpoints of interest, particularly hypothyroidism and atopy, which require provocative testing for confirmation. We are currently collaborating with veterinary experts to develop an adjudication strategy for secondary endpoints of concern.

General limitations of cohort and questionnaire-based studies are also considerations for GRLS. As data collection is annual, respondents may have difficulty with recall or become fatigued due to the questionnaire length. Additionally, since this study has been ongoing for almost a decade, there have been significant changes in commercial dog food companies, pharmaceuticals, and other variables of interest that can make accurately categorizing data more difficult. Preferred laboratory techniques have changed and expanded over time and the Study sampling and preservation methods are not compatible with all of them.

## Can I get hold of the data? where can I find out more?

Select data are publicly available to researchers affiliated with a university, non-profit, or government agency through the Morris Animal Foundation Data Commons site (https://datacommons.morrisanimalfoundation.org/). New data is typically uploaded within 12 months of the completion of a study year. We are continuously working to increase the amount of data available on Data Commons and plan to ultimately include all non-identifying information and genotyping data.

In addition, both academic and private sector researchers can apply to access additional data and/or biospecimens through our request for proposal process. More information is available at: https://www.morrisanimalfoundation.org/golden-retriever-lifetime-study-rfp.

## Supporting information

S1 Fig Participant compliance and retention for baseline through study year six. Dogs were considered fully compliant if we received a completed Annual Owner Questionnaire, biospecimen samples, and Annual Veterinarian Questionnaire for a given study year. Due to difficulties scheduling veterinary appointments during the COVID-19 pandemic, there was an increase in the number of dogs who were partially compliant during study years 5 and 6.
(TIF)

S2 Fig Summary of diagnostic tiers for primary endpoints. (PDF)

S1 Table Detailed information on annual tests conducted at diagnostic laboratories. Laboratory changeover occurred on December 1, 2020.
(PDF)

S2 Table Diagnostic criteria for cancer tiers of confidence. (PDF)

S1 Data (CSV)
