# Supplementary figures and images for: Cohort profile: The Golden Retriever Lifetime Study (GRLS)
Source: PLoS One. 2022 Jun 9;17(6):e0269425. doi: 10.1371/journal.pone.0269425 (PMC9182714; doi:10.1371/journal.pone.0269425)

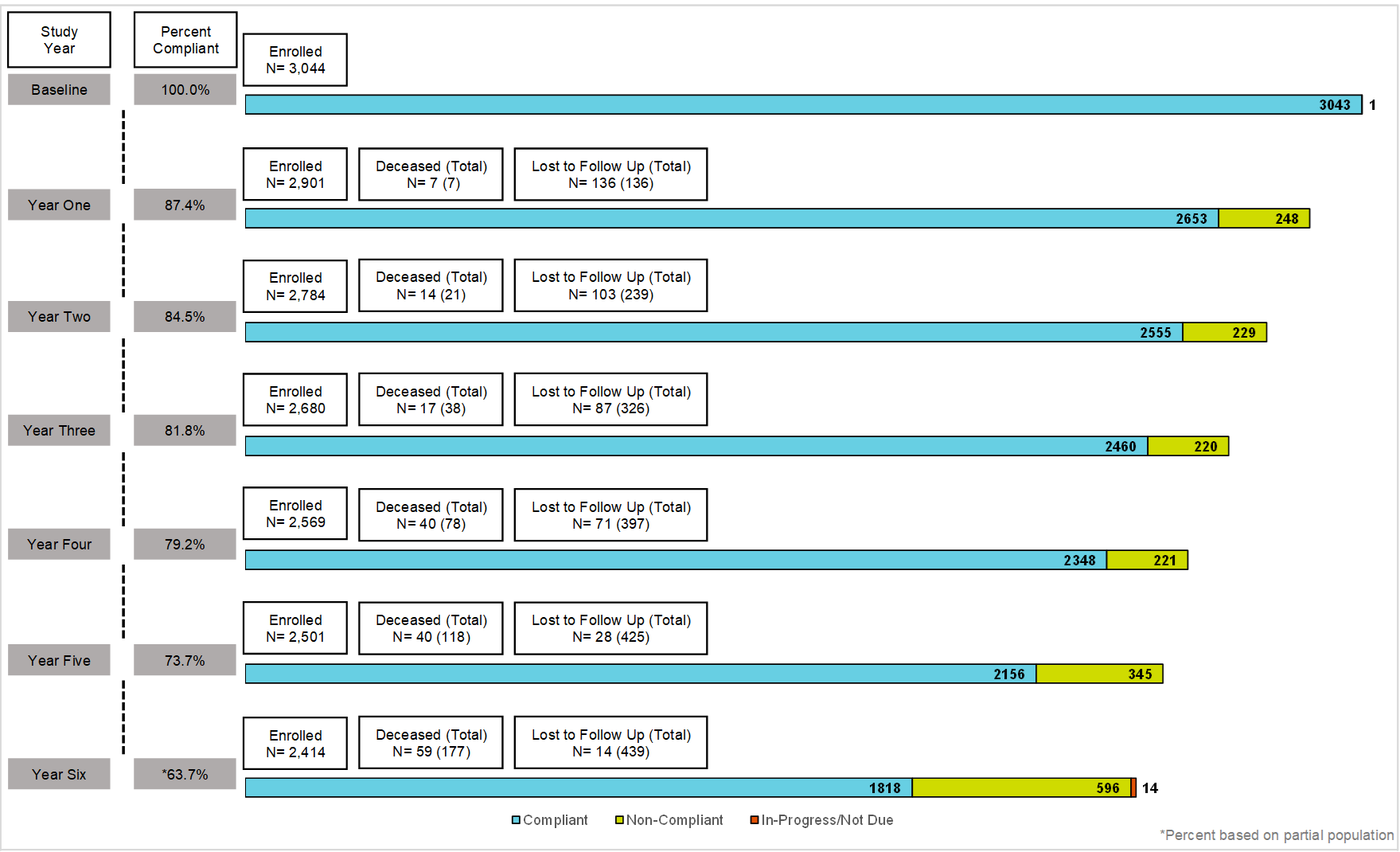

Supplement: S1 Fig — Dogs were considered fully compliant if we received a completed Annual Owner Questionnaire, biospecimen samples, and Annual Veterinarian Questionnaire for a given study year. Due to difficulties scheduling veterinary appointments during the COVID-19 pandemic, there was an increase in the number of dogs who were partially compliant during study years 5 and 6. (TIF) [file pone.0269425.s001.tif]

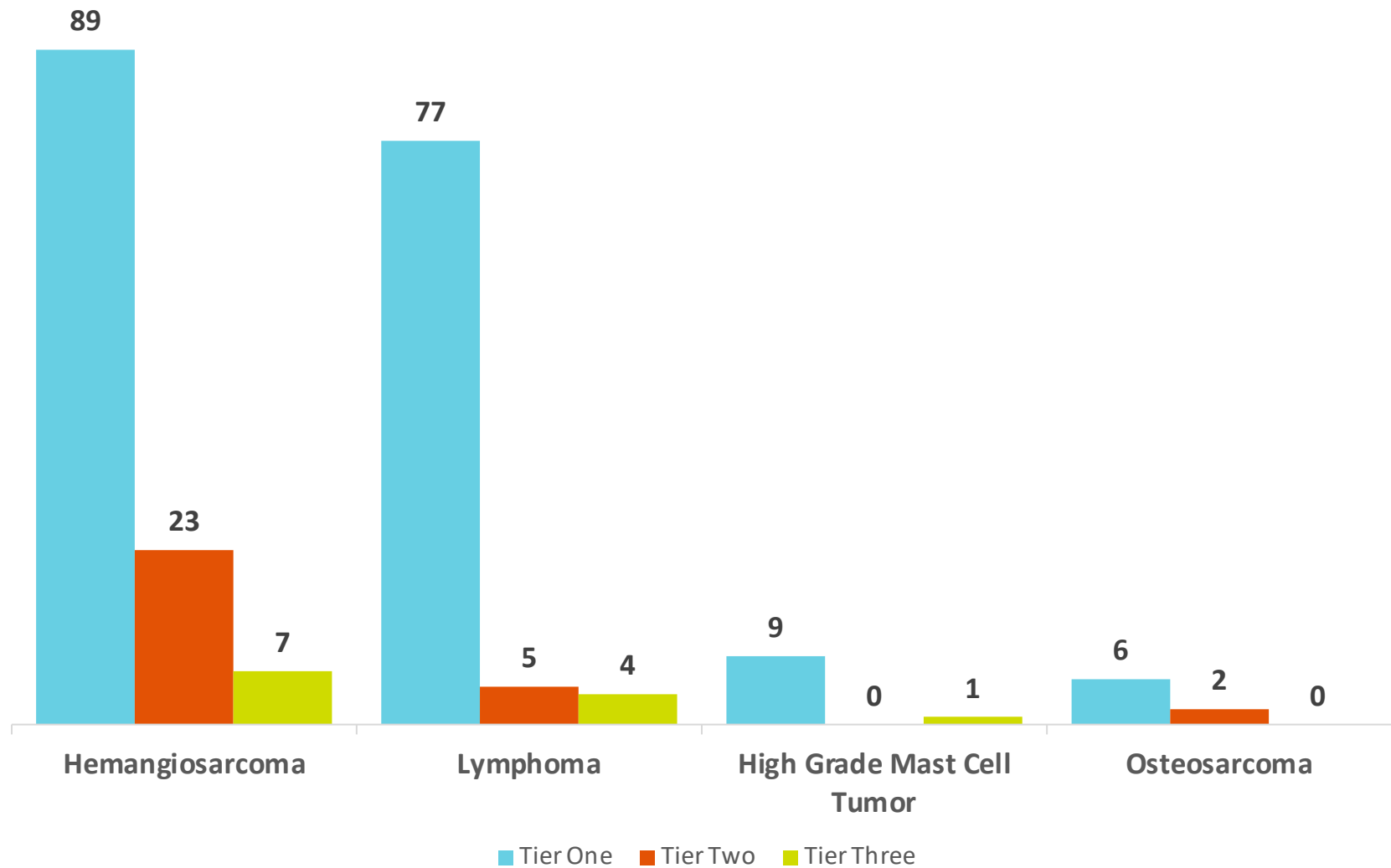

Supplement: S2 Fig — (PDF) [file pone.0269425.s002.pdf]
