# Supplementary material for: Cohort profile: The Golden Retriever Lifetime Study (GRLS)
Source: PLoS One. 2022 Jun 9;17(6):e0269425. doi: 10.1371/journal.pone.0269425 (PMC9182714; doi:10.1371/journal.pone.0269425)
Supplement: S1 Table — Laboratory changeover occurred on December 1, 2020. (PDF) [file pone.0269425.s003.pdf]

	Initial Diagnostic Laboratory	Current Diagnostic Laboratory
**CHEMISTRY**		
Instrument used	Beckman Coulter AU-series	Beckman Coulter AU-series
Glucose	✓	✓
Creatinine	✓	✓
BUN	✓	✓
BUN: Creatinine Ratio	✓	✓
Phosphorus	✓	✓
Calcium	✓	✓
Corrected Calcium	✓	-
Magnesium	✓	✓
Sodium	✓	✓
Potassium	✓	✓
Sodium: Potassium Ratio	✓	✓
Chloride	✓	✓
CO2	-	✓
Anion Gap	-	✓
Osmolality (Calc)	-	✓
Total Protein	✓	✓
Albumin	✓	✓
Globulin	✓	✓
Albumin: Globulin Ratio	✓	✓
ALT	✓	✓
AST	✓	✓
Alk Phosphatase	✓	✓
GGT	✓	✓
Total Bilirubin	✓	✓
Cholesterol	✓	✓
Creatinine Kinase (CK)	✓	✓
Triglyceride	✓	-
Amylase	✓	-
**COMPLETE BLOOD COUNT**		
Instrument used	Siemens Advia	Siemens Advia
WBC	✓	✓
RBC	✓	✓
Hemoglobin (HGB)	✓	✓
Hematocrit (HCT)	✓	✓
MCV	✓	✓
MCHC	✓	✓
CHCM	-	✓
RDW	-	✓
Neutrophil - Count and %	✓	✓
Lymphocyte - Count and %	✓	✓
Monocyte - Count and %	✓	✓
Eosinophil - Count and %	✓	✓
Basophil - Count and %	✓	-
Platelet Count	✓	✓
Platelet Estimate	✓	-

MPV	-	✓
**URINALYSIS**		
Instrument used	Siemens Atlas	Siemens Novus
Color	✓	✓
Clarity/appearance	✓	✓
Specific Gravity	✓	✓
Glucose	✓	✓
Bilirubin	✓	✓
Ketones	✓	✓
Occult blood	✓	✓
pH	✓	✓
Protein	✓	✓
WBCs	✓	✓
RBCs	✓	✓
Epithelial Cells	✓	✓
Bacteria	✓	✓
Crystals	✓	✓
Casts	✓	✓
**PARASITOLOGY**		
Ova & Parasite	Zinc sulfate float	Sugar float
Heartworm antigen	Accuplex Immunoassay	Dirocheck ELISA
**ADDITIONAL TESTS**		
Total T4	Beckman Coulter AU-series	Siemens Immunolite 2000 XPI
Pancreatitis assay	PrecisionPSL	DGGR Lipase
