# Supplementary material for: Cohort profile: The Golden Retriever Lifetime Study (GRLS)
Source: PLoS One. 2022 Jun 9;17(6):e0269425. doi: 10.1371/journal.pone.0269425 (PMC9182714; doi:10.1371/journal.pone.0269425)
Supplement: S2 Table — (PDF) [file pone.0269425.s004.pdf]

Confidence	Tier	Category
Definitive	1	Microscopically confirmed (histology or cytology*)
Presumptive	2	Not microscopically confirmed, but strongly suspected due to direct visualization or imaging^
	3	Clinical suspicion only

*Cytology must be reviewed by a DACVP, otherwise tier 2

^imaging must be reviewed by a DACVR, otherwise tier 3
